# Demographic and geographic disparities in mortality among U.S. adults with osteomyelitis: a CDC WONDER analysis, 1999–2023

**DOI:** 10.1186/s12879-026-13205-w

**Published:** 2026-08-03

**Authors:** Cinthiya Chander, John Paul Braun, Vikram Murugan, Erica J. Stohs, Abubakar Tauseef

**Affiliations:** 1 Creighton University School of Medicine, Omaha, NE USA; 2 Department of Infectious Disease, Creighton University School of Medicine, Omaha, NE USA; 3 Department of Internal Medicine, Creighton University School of Medicine, Omaha, NE USA

**Keywords:** Age-adjusted mortality, Chronic infection, Bone infection, Health inequities, Temporal trends, Rural-urban differences

## Abstract

**Introduction:**

Osteomyelitis, an infection of the bone and bone marrow, causes substantial morbidity and mortality, particularly in older adults and those with comorbidities such as diabetes and peripheral vascular disease. This study examines national trends in mortality among patients with osteomyelitis from 1999 to 2023 using CDC WONDER. Despite therapeutic advances, U.S. mortality is rising, with significant demographic and geographic disparities.

**Methods:**

Using CDC WONDER Multiple Cause-of-Death data, we identified U.S. adults ≥ 35 years with osteomyelitis (ICD-10 M86) as a contributing cause of death. Age-adjusted mortality rates (AAMRs) were calculated using the 2000 U.S. standard population, and trends analyzed with Joinpoint regression to estimate annual and average annual percent change (APC, AAPC) with 95% CIs.

**Results:**

From 1999 to 2023, 111,115 deaths among patients with osteomyelitis occurred. AAMR rose from 1.7 to 4.1 (AAPC: 3.6%), peaking in 2018–2021 (APC: 13.2%). Males had higher rates than females (2023 AAMR: 5.7 vs. 2.9), but both increased proportionally. American Indian/Alaska Native individuals had the highest 2023 rate (8.0) and largest post‑2010 rise, followed by Black individuals (7.1). Mortality increased with age, reaching 33.6 in those ≥ 85 years. Rural areas saw faster increases than urban areas, surpassing them by 2020.

**Conclusions:**

Mortality among patients with osteomyelitis in U.S. adults more than doubled since 1999, accelerating sharply in the last decade and during the COVID‑19 pandemic. Disproportionate burdens among males, AI/AN and Black populations, the oldest adults, and rural residents highlight the need for targeted, equity‑focused interventions and improved access to preventive and specialty care.

## Introduction

Osteomyelitis, an infection of bone and bone marrow, remains a significant clinical challenge with substantial morbidity and mortality, particularly among older adults and those with chronic comorbidities such as diabetes mellitus and peripheral vascular disease [1, 2]. Despite advances in antimicrobial therapy and surgical management, osteomyelitis continues to contribute to substantial mortality, especially in the context of an aging population, rising prevalence of diabetes, and increasing rates of antimicrobial resistance [3, 4].

Recent epidemiological studies suggest that osteomyelitis disproportionately affects certain demographic groups, with notable demographic disparities seen in age, sex, and geography [2, 5]. However, comprehensive national-level data on long-term trends in mortality among patients with osteomyelitis in the United States remain limited.

This study utilizes data from the Centers for Disease Control and Prevention Wide-ranging Online Data for Epidemiologic Research (CDC WONDER) database to provide a detailed analysis of mortality in patients with osteomyelitis in the United States from 1999 to 2023. By elucidating these temporal patterns across demographic and geographic groups, we aim to inform future public health strategies and clinical interventions to reduce the burden of osteomyelitis and address persistent health disparities.

## Methods

### Study design and database

This was a retrospective population-based mortality trend analysis using CDC WONDER data from 1999 to 2023. The study period of 1999–2023 was selected because 1999 was the first full year of U.S. mortality data coded using ICD-10, and 2023 was the most recent year of finalized, complete mortality data available in CDC WONDER at the time of analysis. CDC WONDER was used to identify deaths among patients diagnosed with osteomyelitis occurring within the United States [6]. Death certificate data from the Multiple Cause-of-Death Public Use Record were analyzed to determine deaths among patients diagnosed with osteomyelitis. This database has previously been used in several other studies to analyze nationwide trends in mortality of emphysema [7]. Mortality among patients diagnosed with osteomyelitis was identified using the International Classification of Diseases, 10th Revision, Clinical Modification codes M86 in patients ≥ 35 years. Individuals younger than 35 years were excluded because osteomyelitis-related deaths were rare in this age group among every year studied, resulting in small annual cell counts that precluded stable age-adjusted mortality rate estimation and reliable trend analysis. This study was exempt from institutional review board approval because of the CDC WONDER database containing anonymized, publicly available data.

### Demographic and geographical study groups

We extracted mortality among patients diagnosed with osteomyelitis and population data from 1999 to 2023. Data on demographic and regional groups were extracted, including gender, race/ethnicity, age, urban-rural classification, region, and states. Racial/ethnicity groups were defined as non-Hispanic (NH) White, NH Black, NH American Indian/Alaskan Native, NH Asian/Pacific Islander, and Hispanic people as identified on death certificates. Age groups were defined as 25–39, 40–54, 55–69, 70–84, 85 + years of age. Urban-rural classification followed the 2013 National Center for Health Statistics Urban-Rural Classification Scheme to divide the population into urban (large metropolitan area [population ≥ 1 million], medium/small metropolitan area [population 50,000 to 999,999]) and rural (population < 50,000) counties per the 2013 United States census classification [6]. Urban–rural analyses were restricted to 1999–2020 because post-2020 changes in classification limited comparability across years. Regions were classified into Northeast, Midwest, South, and West according to the Census Bureau definitions [6].

### Statistical analysis

Crude and age-adjusted mortality rates (AAMR) per 100,000 US adults among patients diagnosed with osteomyelitis above the age of 35 were calculated. Crude mortality rates were calculated by dividing the number of deaths among patients diagnosed with osteomyelitis by the corresponding United States population. AAMR were standardized using the 2000 United States standard population as previously described. Temporal trends from 1999 to 2023 were assessed using the Joinpoint Regression Program (version 5.4.0) developed by the National Cancer Institute [8]. Joipoint regression identifies statistically significant changes in temporal trends by fitting a series of connected linear segments to data where significant variation occurred. The software evaluates models with varing number of joinpoints and determines the optimal model using Monte Carlo permutation testing, which assesses whether the addition of joinpoints significantly improves model fit. Because this approach is data-driven, the number and location of joinpoints may vary across demographic groups depending on where statistically significant inflection points in morality trends occur. Annual percentage change (APC) with 95% confidence intervals (CIs) for the AAMRs were calculated for each segment using the Monte Carlo permutation test. The average annual percent change (AAPC) was computed as a weighted average of APCs over the full study period. APC and AAPC values were considered statistically significant if their 95% CIs did not include zero using a 2-tailed t-test, and a p-value ≤ 0.05 was used for significance testing.

## Results

### Mortality among patients with osteomyelitis overall

**Fig. 1:**
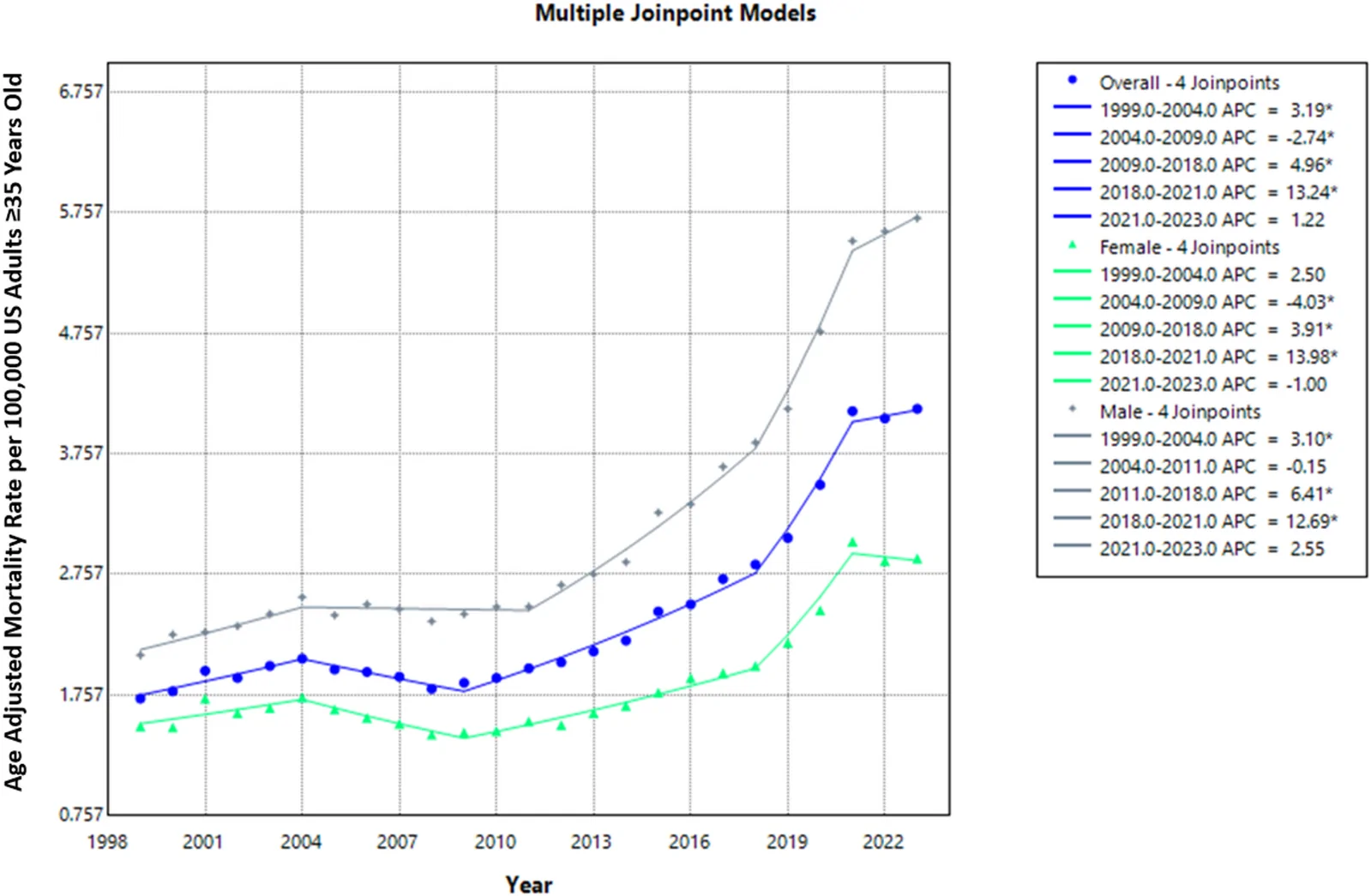
Overall mortality among patients diagnosed with osteomyelitis

**Table 1:** Mortality among patients diagnosed with osteomyelitis AAPC values of investigated demographic factors

Demographic Factor	Cohort	Lower Endpoint	Upper Endpoint	AAPC (95% CI)	*P*-Value
Overall	Full Range	1999	2023	3.6114* (2.6006, 4.632)	< 0.000001
	Female	1999	2023	2.6845* (1.2444, 4.1451)	0.000237
	Male	1999	2023	4.1932* (3.2228, 5.1726)	< 0.000001
Region	Northeast	1999	2023	3.0286* (1.7372, 4.3365)	0.000004
	Midwest	1999	2023	3.6649* (2.0683, 5.2865)	0.000005
	South	1999	2023	3.8198* (2.7532, 4.8975)	< 0.000001
Race	Asian or Pacific Islander	1999	2023	1.4127 (-0.307, 3.1621)	0.107922
	Black or African American	1999	2023	2.9537* (2.1407, 3.7732)	< 0.000001
	White	1999	2023	4.0420* (2.8797, 5.2175)	< 0.000001
Urban/Rural	Urban	1999	2020	2.8802* (1.8659, 3.9045)	< 0.000001
	Rural	1999	2020	5.1110* (3.4264, 6.8229)	< 0.000001
Age Group	35–44 years	1999	2023	5.9408* (3.8902, 8.0318)	< 0.000001
	45–54 years	1999	2023	5.3222* (4.4385, 6.2134)	< 0.000001
	55–64 years	1999	2023	5.3882* (4.5425, 6.2407)	< 0.000001
	65–74 years	1999	2023	4.8154* (3.0677, 6.5929)	< 0.000001
	75–84 years	1999	2023	3.4124* (2.7768, 4.0519)	< 0.000001
	85 + years	1999	2023	1.8740* (1.1206, 2.6331)	0.000001

Between 1999 and 2023 there were 111,115 deaths in U.S. adults aged 35 years or older diagnosed with osteomyelitis (Fig. 1). Overall age-adjusted mortality rates (AAMR) increased during this period from 1.7* (95% CI 1.6 to 1.8) in 1999 to 4.1* (95% CI 4.0 to 4.2) in 2023, with an average annual percentage change (AAPC) of 3.6* (95% CI 2.6 to 4.6) (Table 1). The annual percent change (APC) in AAMR was 3.2* from 1999 to 2004 (95% CI 1.0 to 5.4), which decreased to -2.7* (95% CI -5.3 to -0.1) from 2004 to 2009, increased to 5.0* (95% CI 4.2 to 5.8) from 2009 to 2018, increased again to 13.2* (95% CI 7.1 to 19.7) from 2018 to 2021, and decreased to 1.2 (95% CI -3.2 to 5.8) from 2021 to 2023.

### Mortality among patients with osteomyelitis by gender

Between 1999 and 2023 there were 62,519 deaths in males aged 35 years or older diagnosed with osteomyelitis in the United States (Fig. 1). Overall AAMR increased during this period from 2.1* (95% CI 2.0 to 2.2) in 1999 to 5.7* (95% CI 5.6 to 5.9) in 2023, with an AAPC of 4.2* (95% CI 3.2 to 5.2) (Table 1). The APC in AAMR was 3.1* from 1999 to 2004 (95% CI 0.9 to 5.4), which decreased to -0.2 (95% CI -1.5 to 1.3) from 2004 to 2011, increased to 6.4* (95% CI 5.2 to 7.7) from 2011 to 2018, increased again to 12.7* (95% CI 6.8 to 18.9) from 2018 to 2021, and decreased to 2.5 (95% CI -2.3 to 7.6) from 2021 to 2023.

Between 1999 and 2023 there were 48,596 deaths in females aged 35 years or older diagnosed with osteomyelitis in the United States (Fig. 1). Overall AAMR increased during this period from 1.5* (95% CI 1.4 to 1.6) in 1999 to 2.9* (95% CI 2.8 to 3.0) in 2023, with an AAPC of 2.7* (95% CI 1.2 to 4.1) (Table 1). The APC in AAMR was 2.5 (95% CI 0.0 to 5.1) from 1999 to 2004, which decreased to -4.0* (95% CI -7.4 to -0.5) from 2004 to 2009, increased to 3.9* (95% CI 2.7 to 5.1) from 2009 to 2018, increased again to 14.0* (95% CI 4.8 to 24.0) from 2018 to 2021, and decreased to -1.0 (95% CI -8.2 to 6.8) from 2021 to 2023.

### Mortality among patients with osteomyelitis by race

**Fig. 2:**
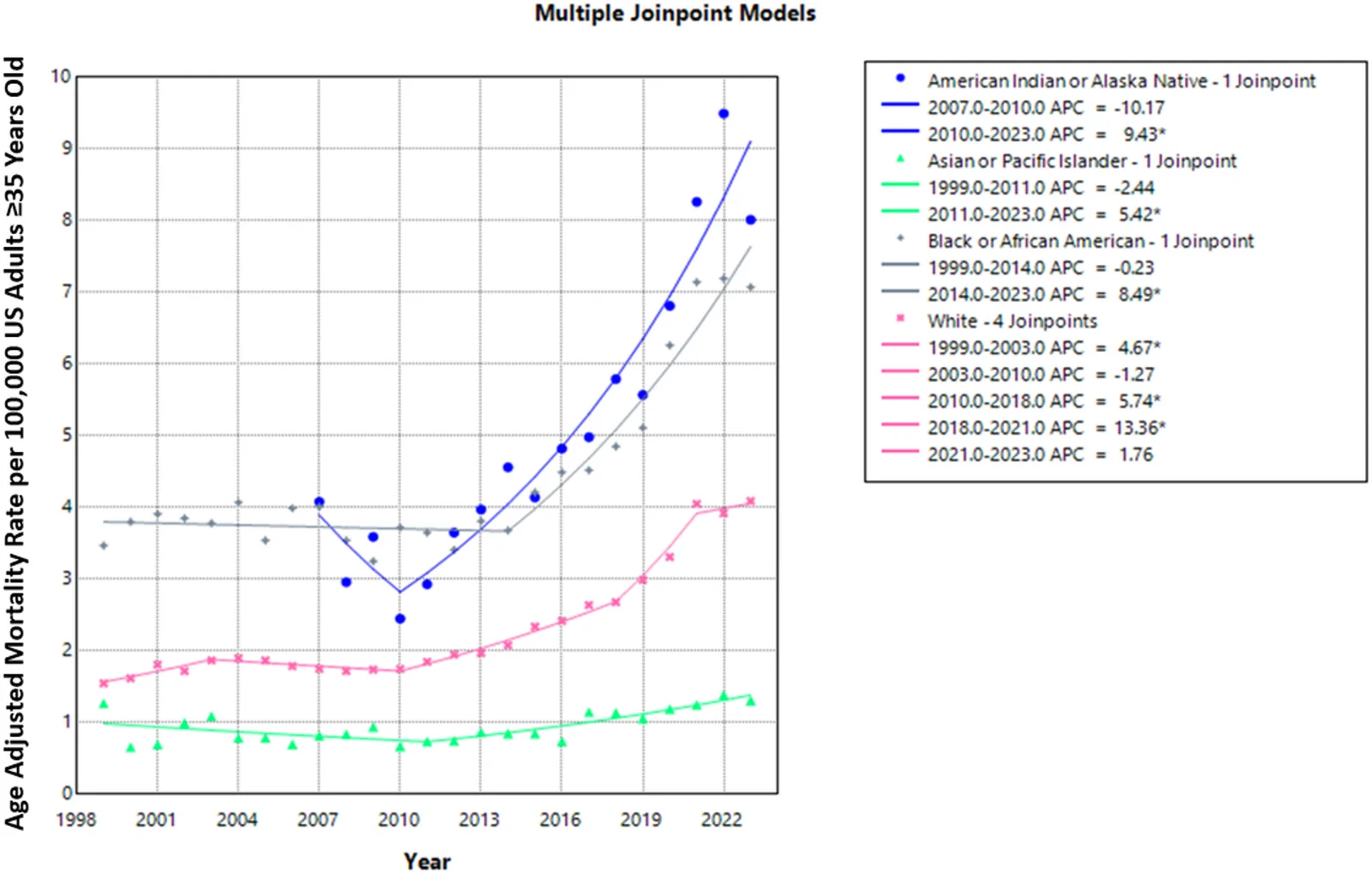
Mortality among patients diagnosed with osteomyelitis by race

American Indian or Alaska Native (AI/AN) individuals had the highest AAMR in 2007 with a value of 4.0* (95% CI 2.7 to 5.9), and the highest AAMR in 2023 with a value of 8.0* (95% CI 6.4 to 9.6) (Fig. 2). The APC was − 10.2 from 2007 to 2010 and subsequently increased to 9.4* from 2010 to 2023.

Black or African American individuals had the highest AAMR in 1999 with a value of 3.5* (95% CI 3.1 to 3.8) and the second-highest AAMR in 2023 with a value of 7.1* (95% CI 6.7 to 7.4) (Fig. 2). The APC was − 0.2 from 1999 to 2014 and subsequently increased to 8.5* from 2014 to 2023.

White individuals had the second-lowest AAMR for every year studied, with 1.6* (95% CI 1.5 to 1.6) in 1999 and 4.1* (95% CI 4.0 to 4.2) in 2023 (Fig. 2). The APC was 4.7* from 1999 to 2004, decreased to -1.3 from 2003 to 2010, increased to 5.7* from 2010 to 2018, increased to 13.4*, and tapered off to 1.8 from 2021 to 2023.

Asian or Pacific Islander individuals had the lowest AAMR for every year studied (and experienced the smallest increase in AAMR), with 1.3* (95% CI 0.9 to 1.8) in 1999 and 1.3* (95% CI 1.1 to 1.5) in 2023 and AAPC of 1.4 (Fig. 2; Table 1). The APC was − 2.4 from 1999 to 2011 and 5.4* from 2011 to 2023.

### Mortality among patients with osteomyelitis by age (≥ 35)

**Fig. 3:**
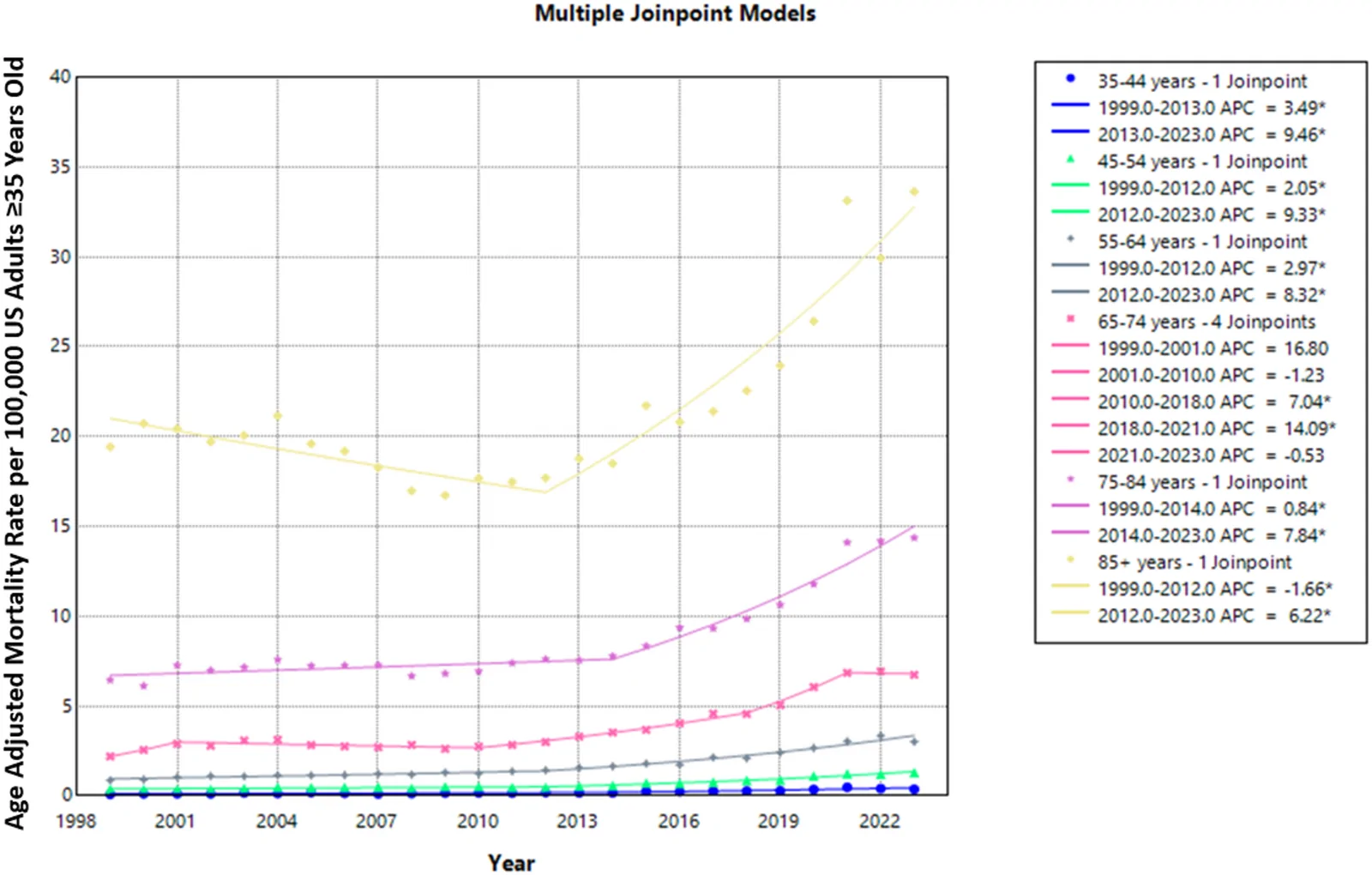
Mortality among patients diagnosed with osteomyelitis by age

The 85 + year category had the highest crude mortality rate for every year studied, with a value of 19.4 in 1999 and 33.6 in 2023 (Fig. 3). They had an AAPC of 1.9* (Table 1). Their APC was − 1.7* from 1999 to 2012 and increased to 6.2* from 2012 to 2023.

The 75–84 year age category had a crude mortality rate of 6.5 in 1999, 14.4 in 2023, and an AAPC of 3.4* (Fig. 3; Table 1). Their APC was 0.8* from 1999 to 2014 and increased to 7.8* from 2014 to 2023.

The 65–74 year age category had a crude mortality rate of 2.2 in 1999, 6.7 in 2023, and an AAPC of 4.8* (Fig. 3; Table 1). Their APC was 16.8 from 1999 to 2001, decreased to -1.2 from 2001 to 2010, increased to 7.0*, increased again to 14.1* from 2018 to 2021, and decreased to -0.5 from 2021 to 2023.

The 55–64 year age category had a crude mortality rate of 0.9 in 1999, 3.0 in 2023, and an AAPC of 5.4* (Fig. 3; Table 1). Their APC was 3.0* from 1999 to 2012 and increased to 8.3* from 2012 to 2023.

The 45–54 year age category had a crude mortality rate of 0.4 in 1999, 1.3 in 2023, and an AAPC of 2.0* (Fig. 3; Table 1). Their APC was 2.1* from 1999 to 2012 and increased to 8.3* from 2012 to 2023.

The 35–44 year age category had the lowest crude mortality rate for every year studied, with a value of 0.1 in 1999, 0.4 in 2023, and an AAPC of 3.4* (Fig. 3; Table 1). Their APC was 3.5* from 1999 to 2013 and increased to 9.5* from 2013 to 2023.

### Mortality among patients with osteomyelitis by urban vs. rural

**Fig. 4:**
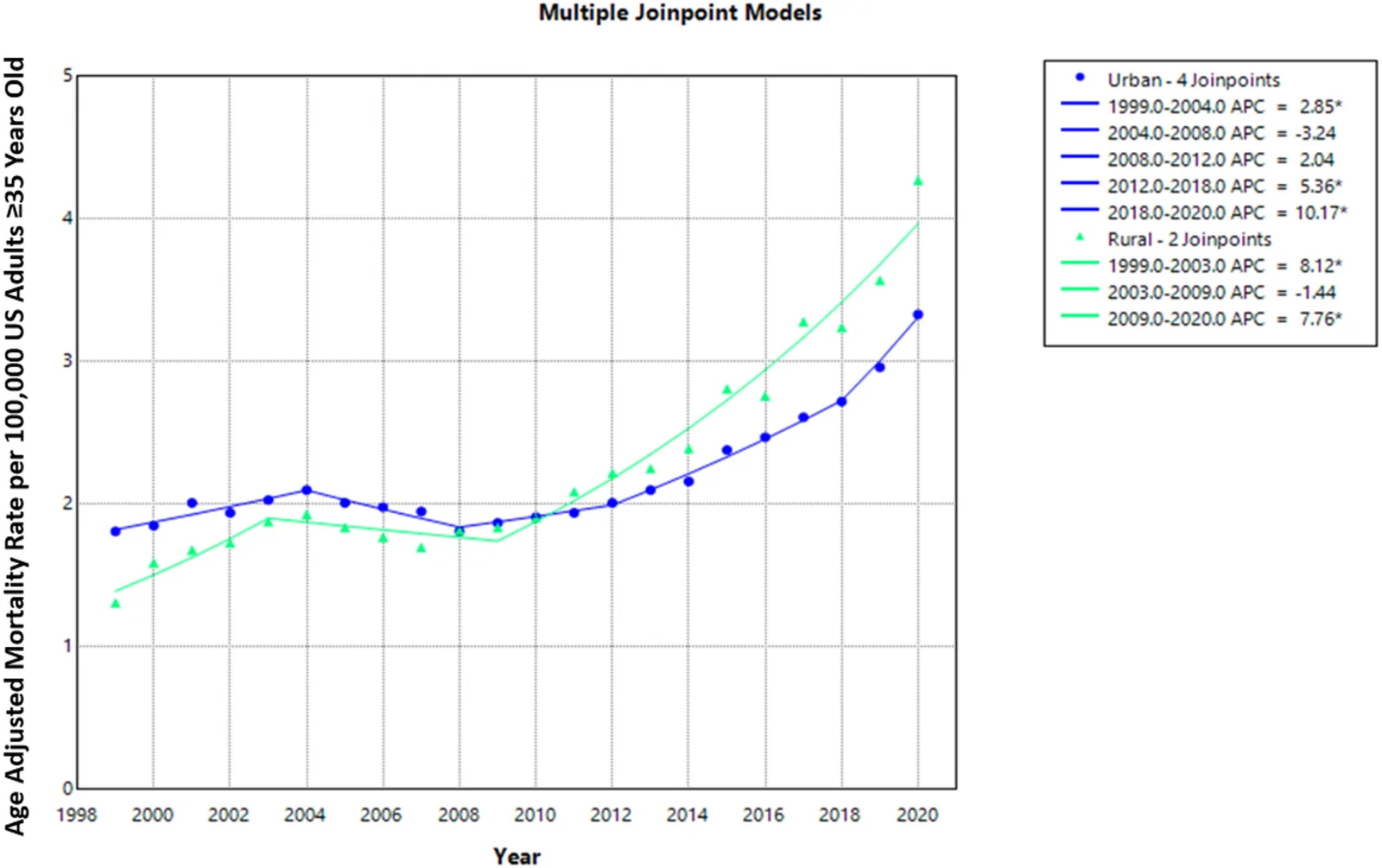
Mortality among patients diagnosed with osteomyelitis by urban vs. rural

Urban regions initially had an AAMR of 1.8 in 1999, which was higher than rural regions (Fig. 4). However, urban regions experienced a smaller overall increase and had a smaller AAMR in 2020 with a value of 3.3. The APC in urban regions was 2.9* from 1999 to 2004, decreased to -3.2 from 2004 to 2008, increased to 2.0 from 2008 to 2012, increased to 5.4* from 2012 to 2018, and increased again to 10.2* from 2018 to 2020. The AAPC in urban regions was 2.9* (Table 1).

In contrast, rural regions had a smaller AAMR in 1999 with a value of 1.3 but increased to a larger AAMR in 2020 with a value of 4.3 (Fig. 4). The APC was 8.1* from 1999 to 2003, decreased to -1.4 from 2003 to 2009, and increased to 7.8* from 2009 to 2020. The AAPC in rural regions was 5.1* (Table 1).

## Discussion

### Overall mortality

Previous research on mortality among patients with osteomyelitis trends in the United States has been limited, with most studies focusing on specific populations or subtypes rather than national surveillance data [1, 2]. The observed increase in mortality among patients with osteomyelitis rates can be attributed to several converging factors.

First, the aging population in the United States has created a larger cohort of individuals with predisposing conditions for osteomyelitis. Older adults are particularly vulnerable to osteomyelitis due to increased incidence of associated disorders such as peripheral vascular disease, diabetes mellitus, malnutrition, and compromised immune function [9, 10, 11, 12].

Second, the diabetes epidemic has played a crucial role in the rising mortality among patients with osteomyelitis rates. Diabetes-related osteomyelitis has shown particularly concerning trends, with age-adjusted mortality rates increasing from 2.63 per 1,000,000 person-years in 1999 to 4.25 per 1,000,000 person-years in 2017 [11]. Patients with diabetes may have a 15–25% lifetime risk of developing a foot ulcer, with 20% of infections progressing to osteomyelitis. The mortality associated with diabetic foot ulcers, which frequently develop osteomyelitis, is substantial, with 5-year mortality rates ranging from 50 to 70% [13]. However, because the CDC WONDER mortality dataset does not contain population-level prevalence data for diabetes or other comorbidities, the present study cannot speculate whether the observed increase in mortality among patients with osteomyelitis represents an independent rise in osteomyelitis incidence or partially reflects increasing diabetes prevalence in the United States.

Third, the emergence of antibiotic-resistant organisms has significantly complicated treatment outcomes. Studies have shown that chronic osteomyelitis is increasingly caused by multidrug-resistant organisms, with some antibiotics promoting the formation of small colony variants that enhance bacterial persistence and treatment resistance [14]. The proportion of methicillin-resistant Staphylococcus aureus (MRSA) infections has increased, with some Native American populations experiencing rates over 75% [15].

### Mortality by gender

This study’s finding that males have consistently higher osteomyelitis mortality rates (AAPC 4.2% vs. 2.7% in females) aligns with established patterns in infectious disease mortality (Fig. 1). Males accounted for 62,519 of the total 111,115 deaths (56.3%) despite similar population sizes. This gender disparity is consistent with broader infectious disease patterns, where males typically experience higher mortality rates [16].

The higher osteomyelitis mortality observed in males may be multifactorial. Males have a higher prevalence of several established risk factors for osteomyelitis, including diabetes mellitus and tobacco use, both of which are associated with impaired wound healing and increased susceptibility to infection [17, 18]. In addition, males are more likely to be employed in occupations involving manual labor or trauma-prone environments, which may increase the risk of skin and soft tissue injuries that can predispose to bone infection [19]. Prior literature also suggests that males are less likely to engage in preventive healthcare and may delay seeking medical attention, potentially contributing to more advanced disease at presentation [20, 21]. However, sex-based differences in disease severity and presentation remain complex and may vary by condition, highlighting the need for further investigation into biological, behavioral, and healthcare access factors underlying these disparities.

### Mortality by race

American Indian/Alaska Native (AI/AN) individuals had the highest mortality among patients with osteomyelitis rates by 2023 (8.0 per 100,000), representing a concerning health disparity (Fig. 2). Prior to 2010, AI/AN populations had lower rates than Black populations but experienced the largest increases over the study period (APC of 9.4% from 2010 to 2023). This pattern reflects broader health disparities experienced by Native American populations, who have significantly higher rates of diabetes, invasive bacterial infections, and other conditions predisposing to osteomyelitis [22]. Additionally, healthcare access barriers, including geographic isolation and underfunding of Indian Health Service facilities, may contribute to delayed diagnosis and treatment [22].

Black individuals consistently maintained high mortality among patients with osteomyelitis rates throughout the study period, with rates of 7.1 per 100,000 in 2023 (Fig. 2). These disparities reflect well-documented racial inequities in healthcare access and outcomes including higher rates of diabetes, limited healthcare access, and structural racism affecting quality of care [23].

Asian/Pacific Islander individuals had the lowest mortality among patients with osteomyelitis rates throughout the study period and experienced the smallest increases (Fig. 2). This pattern likely reflects the increased access to healthcare among Asian individuals, reflected by their increased rates of insurance [24]. However, recent research has highlighted that Pacific Islander populations, when disaggregated from Asian populations, experience significantly higher mortality rates across multiple conditions [25]. This suggests that combining these diverse populations may mask important health disparities within Pacific Islander communities.

### Mortality by age

The stepwise increase in mortality among patients with osteomyelitis rates with advancing age reflects well-established patterns in infectious disease outcomes (Fig. 3). The finding that individuals aged 85 + years had the highest crude mortality rates (33.6 per 100,000 in 2023) is consistent with age-related declines in immune function and increasing comorbidity burden [3].

The elderly population (85 + years) experienced substantial increases in mortality rates, particularly after 2012 (APC of 6.2% from 2012 to 2023) (Fig. 3). This trend reflects several concerning factors. First, the growing elderly population with multiple comorbidities creates a larger pool of individuals at high risk for osteomyelitis. Second, elderly patients with osteomyelitis have significantly higher rates of complications and treatment failures [8].

The accelerated mortality increases in recent years among elderly patients may also reflect the impact of prosthetic joint infections, which are increasingly common in this age group. Studies have shown that prosthetic joint infections carry mortality rates of 5.5% at one year and 7.3% at two years, with higher rates in elderly patients [26]. The growing number of joint replacement surgeries in elderly patients creates an expanding population at risk for these serious infections.

### Mortality by urban vs. rural

Rural populations experienced larger increases in mortality among patients diagnosed with osteomyelitis and ultimately surpassed urban population rates (Fig. 4), a finding consistent with research on other diseases. Studies of diabetes-related complications, which frequently lead to osteomyelitis, have shown that rural populations experience higher rates of complications and poorer outcomes [27]. Rural patients also face significant barriers including limited access to specialty care, longer travel distances to healthcare facilities, and reduced availability of multidisciplinary care teams necessary for complex infections [28].

### Implications for osteomyelitis diagnosis and management

The demographic and geographic disparities in mortality among patients with osteomyelitis observed in this study likely reflect differences in the timeliness of diagnosis and access to specialized care. Osteomyelitis is often underrecognized in high-risk populations, particularly among individuals with diabetes, peripheral vascular disease, and chronic wounds, leading to delayed imaging and advanced disease at presentation [28]. Prior studies show that delayed diagnosis is associated with higher rates of chronic infection, amputation, and mortality, whereas early imaging and multidisciplinary management improve outcomes [13, 25]. The higher mortality observed among rural populations and American Indian/Alaska Native individuals suggests that limited access to coordinated specialty care may contribute to these disparities. Strategies aimed at improving early detection and expanding access to multidisciplinary care in underserved populations may help reduce osteomyelitis-related mortality disparities.

### Strengths & limitations

This study has several strengths. It utilizes a large, nationwide dataset from the CDC WONDER Multiple Cause-of-Death database, providing substantial statistical power and a comprehensive overview of mortality among patients with osteomyelitis in the United States over a 24-year period. The breadth of the dataset allows for in-depth temporal trend analysis and facilitates examination of disparities across numerous subgroups, and the use of Joinpoint regression further strengthens this analysis by identifying statistically significant inflection points in mortality trends.

However, there are notable limitations. As a retrospective, population-level analysis, the study relies on mortality data derived from death certificates, which may contain inaccuracies due to misclassification or reporting errors. The use of aggregate data precludes access to individual-level clinical variables, including comorbidities, treatment history, infection severity, or socioeconomic status, which limits the ability to control for important confounding factors. In particular, the database does not provide population-level prevalence data for major risk factors for osteomyelitis, such as diabetes mellitus, preventing direct evaluation of the prevalence of major risk factors in contributing to the increased mortality trends observed. Furthermore, the study design does not allow for causal inference. The findings are not generalizable to younger individuals, as the analysis focused on patients aged 35 and older, in whom osteomyelitis is more commonly observed. Finally, while the study highlights significant trends and disparities, future research should incorporate prospective cohort studies and clinical datasets to evaluate the influence of healthcare access, intervention strategies, and disease management on osteomyelitis outcomes.

## Conclusion

This national temporal analysis reveals a marked rise in mortality among U.S. adults aged ≥ 35 diagnosed with osteomyelitis from 1999 to 2023. The data highlight widespread increases across racial, gender, age, and geographic groups, with American Indian/Alaska Native and Black populations, males, older adults, and rural residents disproportionately affected, which is likely due to structural health inequities, comorbidity burdens, and healthcare access challenges. Contributing factors include an aging population, rising diabetes rates, antimicrobial resistance, and pandemic-related care disruptions. These findings underscore the urgent need for equity-focused, multidisciplinary public health strategies, enhanced infection surveillance, and improved access to preventative and specialty care in underserved communities.

## Data Availability

Publicly available datasets were utilized for the present study. This data can be accessed online: https://wonder.cdc.gov/mcd.html.
